# Immunogenicity of ChAdOx1 (Covishield) Booster Dose in Healthcare Providers: A Pre-Post Study

**DOI:** 10.7759/cureus.46370

**Published:** 2023-10-02

**Authors:** Trupti Meher, Subrat K Pradhan, Shankar P Hatei, Subash C Majhi, Aishwarya Panda, Smriti R Mund, Sanjeeb K Mishra

**Affiliations:** 1 Community Medicine, Veer Surendra Sai Institute of Medical Sciences and Research, Sambalpur, IND; 2 Anesthesia and Critical Care, Shrirama Chandra Bhanja (SCB) Medical College and Hospital, Cuttack, IND; 3 Pediatrics, Veer Surendra Sai Institute of Medical Sciences and Research, Sambalpur, IND; 4 Anesthesiology, Veer Surendra Sai Institute of Medical Sciences and Research, Sambalpur, IND

**Keywords:** vaccines, immunogenicity, secondary immunization, chadox1 ncov-19, covid-19 vaccines

## Abstract

Background

Worldwide, healthcare workers who face a higher risk of contracting coronavirus disease 2019 (COVID-19) were among the first to receive COVID-19 vaccinations. Following the initial two vaccine doses, health experts recommended a third booster shot to enhance protection against the severe acute respiratory syndrome coronavirus 2 (SARS‑CoV‑2) virus. However, limited information about how this booster dose affects antibody levels is available. This study assesses the immune response triggered by the ChAdOx1 (Covishield) booster dose.

Methods

We conducted a before and after study among 132 healthcare providers at a tertiary care hospital in India who had already received their initial COVID-19 vaccine doses and agreed to participate. A booster shot was administered nine months following their second vaccine dose per the prevalent norms. We collected blood samples to measure immunoglobulin-G (IgG) levels against the spike protein's receptor-binding domain of the SARS-CoV-2 virus. These blood samples were taken both when they received the booster shot and one month after the booster. We determined IgG levels using a chemiluminescence microparticle immunoassay.

Result

Among the participants, approximately 54% were females. Regarding occupation, about 36% were doctors, 30% were students, 20% were nursing officers, and the remaining 14% held grade-4 positions. The median age of the participants was 32 years. About 74% had no history of underlying health conditions. Before the booster dose, 29% of the participants tested negative for antibodies. However, all participants developed antibodies following the booster shot, and there was a significant increase in antibody levels, which was statistically meaningful with a p-value of less than 0.0001.

Conclusion

In conclusion, the administration of a booster dose effectively induced seroconversion and significantly increased antibody levels among healthcare providers, enhancing their immunity against COVID-19, essential in the face of a waning immune response to primary series vaccination.

## Introduction

Coronavirus disease 2019 (COVID-19), caused by the severe acute respiratory syndrome coronavirus 2 (SARS-CoV-2), has become a global health crisis. In March 2020, the World Health Organization (WHO) declared it a pandemic. Various COVID-19 vaccines have been developed and rolled out [1]. India initiated its vaccination campaign on January 16, 2021, primarily targeting 30 million healthcare workers dealing directly with COVID-19 patients [2]. India authorized two vaccines initially, Oxford-AstraZeneca's Covishield (Cambridge, UK) and Bharat Biotech's Covaxin (Hyderabad, India) [2]. Vaccines have been crucial in combating the pandemic, potentially preventing around 4.2 million deaths in India from December 8, 2020, to December 8, 2021 [3].

Globally, over 5 billion people have received complete vaccination with the primary series, with around 1 billion in India alone [4]. Notably, 80% of these recipients have received Covishield [4]. Covishield, also known as the ChAdOx1 nCoV-19 vaccine, employs a modified chimpanzee adenovirus as a viral vector [5]. Initially, it was administered in two doses, spaced three to four weeks apart, but later extended to six to eight weeks due to vaccine shortages. Vaccination has proven effective in reducing hospitalizations and fatalities, yet the duration of this protection remains uncertain. A systematic review of 39 studies revealed that vaccine efficacy against COVID-19 infection in the general population ranges from 89-97% for BNT162b2, 92% for ChAdOx1 nCoV-19, and 94% for mRNA-1273. However, the effectiveness drops to 44.1% for ChAdOx1 nCoV-19 and 62.5% for BNT162b2 by week 20 after the second dose [6-8]. The emergence of new SARS-CoV-2 variants has raised concerns about vaccine efficacy and longevity [9], with many studies reporting waning immunity against these variants for authorized vaccines [10-13].

Despite a nationwide vaccination effort, India, like many countries, faced COVID-19 resurgences fueled by more infectious variants and declining immunity after primary vaccination. This occurred even with over 68% of the population fully vaccinated by December 2022 [4]. Consequently, several countries, including the U.S., started offering third-dose booster vaccines toward the end of 2021 to combat waning immunity and new variants [14].

Healthcare providers (HCPs) at high risk for COVID-19 were among the first to receive vaccinations. After the initial two doses, a third booster dose was recommended to enhance protection against the resurgent COVID-19. However, there are limited data on how this booster affects antibody levels. Few population-based studies have explored the immunogenicity of booster doses. This study aims to assess the immunogenic impact of the ChAdOx1 booster dose.

## Materials and methods

Study design and population

We conducted a before-and-after study involving 132 healthcare providers (HCPs) in a tertiary care hospital in India. Our study group comprised HCPs, including doctors, students, paramedics, administrative staff, and sanitation workers, all aged 18 or older, who had received two doses of the Oxford-AstraZeneca ChAdOx1 (Covishield) vaccine at least nine months prior. We excluded individuals taking immunosuppressive drugs, those who had contracted COVID-19 after their second dose, pregnant women, and anyone currently suffering from an acute illness, as they were not eligible for vaccination. We used G-power software (version 3.1.97) to determine the sample size based on findings from a previous study conducted in the same context. Using the paired T-test formula (Wilcoxon signed-rank test for matched pairs) with an estimated effect size of 0.33 [15], α=0.05, and a power of 0.95 to detect the minimum difference, we calculated a sample size of 127. However, our study ultimately included 132 willing participants.

Study tools and techniques

The study variables encompassed age, gender, blood type, occupation, comorbidity status, height and weight (used to calculate BMI), and pre-booster and post-booster immunoglobulin G (IgG) antibody levels. The primary focus was assessing the change in SARS-CoV-2 spike IgG antibody levels as the outcome variable to gauge the immunogenicity of the ChAdOx1 vaccine. Participants with known conditions like diabetes, hypertension, kidney disease, liver disease, and respiratory issues were categorized as having comorbidities. We collected data through a structured questionnaire administered via interviews. Blood samples were drawn for immunogenicity evaluations at the baseline (on the day of vaccination) and approximately one month after the booster dose. All participants voluntarily joined the study after providing written informed consent, adhering to the principles outlined in the Declaration of Helsinki. The study received ethical approval from the Odisha State Research and Ethics Committee, and enrollment commenced on January 10, 2022.

Serological analysis

A blood sample of approximately 3-5 ml was collected from each participant using sterile, aseptic procedures. These samples were then centrifuged at 2500 rpm for 5 minutes and divided into labeled sterile cryotubes, which were stored at -20°C until needed. To quantitatively detect antibodies against the spike protein of SARS-CoV-2, we employed the ARCH SARS-CoV-2 IgG II Quant commercial kit (Abbott Laboratories, Abbott Park, Illinois). This kit uses a chemiluminescence microparticle immunoassay and is designed for qualitative and quantitative assessment of IgG antibodies to SARS-CoV-2 in serum and plasma. It is compatible with the Alinity and ARCHITECT systems (Abbott Laboratories). The measurable range for this assay is reported to be from 21 to 40,000 AU/ml, with values above this range being recorded as 40,000 AU/ml. A positive result is indicated when the measured value equals or exceeds 50 AU/ml. This method exhibits a sensitivity of 91.6% at all time points, 98.3% after more than 14 days post-vaccination, and a specificity of 99.4% [16].

Statistical analysis

We assessed the normality of continuous variables through both graphical and numerical methods. For categorical variables, we presented them by showing their frequency and percentage. To describe the central tendency of data, we used both the mean and median while the data spread was illustrated using confidence intervals (CI) and interquartile range (IQR). To compare the pre-booster and post-booster groups, we employed relevant statistical tests. We considered a two-tailed p-value of less than 0.05 to indicate statistical significance. Graphical representations were generated using GraphPad Prism Version 9.3.1, and our statistical analysis was conducted using Epi Info (Version 7.2.5.0) from the CDC in Atlanta.

## Results

Our research involved 132 study participants, aged 19 to 82 years, with a median age of 32. Approximately 71 of these participants (about 53.8%) were female. The majority of the participants were doctors (47 individuals), followed by students (40) and nursing officers (26). The most prevalent blood types among the participants were O (34.85%) and B (33.33%). Around a quarter of our study participants (25.76%) had one or more underlying health conditions. Moreover, most participants were overweight (36.52%) or obese (42.42%). These participant characteristics are detailed in Table 1.

**Table 1:** Characteristics of the healthcare providers, Veer Surendra Sai Medical Sciences and Research, Sambalpur, Odisha, India, 2022, n = 132

Variable	Frequency (%)
Age (years)	
< 25	40 (30.3%)
25-50	70 (53%)
>50	22 (16.7%)
Gender	
Female	71 (53.8%)
Male	61 (46.2%)
Body mass index (kg/m2 )	
Underweight (<18.5)	6 (4.5%)
Normal weight (18.5-24.9)	67 (50.8%)
Overweight (25-29.9)	46 (34.9%)
Obese (>29.9)	13 (9.8%)
Blood group	
A	33 (25%)
B	44 33.3%)
AB	9 (6.8%)
O	46 (34.9%)
Occupation	
Doctor	47 (35.6%)
Student	40 (30.3%)
Nursing officer	26 (19.7%)
others	19 (14.4%)
Comorbidities	
Yes	34 (25.8%)
No	98 (74.2%)

Before receiving the booster dose, 38 out of 132 participants (approximately 28.79%) were found to have antibody levels below 50 BAU/ml, categorizing them as seronegative. Among these seronegative individuals, the average age was 40, with a standard deviation of 14.55. In this group, 20 participants (approximately 52.64%) were male, and 12 (around 31.58%) had underlying health conditions. Additionally, 15 participants (approximately 39.4%) were overweight or obese. Blood group O was most prevalent among this group, making up 42.11%, followed closely by blood group B at 39.47%. Notably, all participants achieved seroconversion at 30 days following the booster dose. The geometric mean titer (GMT) in this Pre-booster group was 192 BAU/ml (with a confidence interval of 130.31 to 283.79).

**Table 2:** Change in antibody titer one month after booster dose, Veer Surendra Sai Institute of Medical Sciences and Research, n = 132 BAU - binding antibody unit

Variables	Antibody GMT (BAU/ml) Pre-booster dose (95% CI)	Antibody GMT (BAU/ml) one month after booster dose (95% CI)
Age (Years)		
<25 (n=40)	290.27 (212.85, 395.85)	6847.16 (5955.35, 7872.51)
25-50 (n=70)	220.18 (170.10, 285.01)	5578.78 (5000.82, 6223.54)
>50 (n=21)	72.31 (39.47, 132.47)	9208.77 (7782.33, 10896.67)
Sex		
Male (n=61)	155.70 (113.56, 213.46)	6596.08 (5852.96, 7433.55)
Female (n=71)	231.04 (179.83, 296.82)	6459.70 (5814.05, 7177.04)
Occupation		
Doctor (n=47)	121.60 (84.84, 174.29)	7211.28 (6244.08, 8328.29)
Student (n=40)	252.31 (183.78, 346.38)	6453.14 (5679.01, 7332.80)
Nursing officer (n=26)	471.43 (324.61, 684.66)	5738.11 (5013.45, 6567.52)
Others (n=19)	99.67 (56.20, 176.76)	6200.49 (4749.60, 8094.60)
Body mass index (BMI)		
Underweight (n=5)	381 (134.08, 1082.66)	4963 (3535.17, 6967.51)
Normal weight ((n=36)	269.97 (185.09, 393.77)	6394.16 (5585.11, 7321.58)
Overweight (n=35)	101.58 (67.23, 153.49)	7056.77 (5984.76, 8320.80)
Obese (n=56)	217.33 (162.90, 289.95)	6443.84 (5679.45, 7311.12)
Blood Group		
A (n=33)	470.47 (334.60, 661.50)	7024.59 (6004.56, 8217.89)
AB (n=9)	162.56 (54.21, 487.39)	5829.30 (4273.80, 7950.94)
B (n=44)	133.10 (96.51, 183.57)	6366.06 (5558.85, 7427.05)
O (n=46)	149.20 (106.07, 209.87)	6470.18 (5636.59, 7427.05)
Co-morbidity status		
Yes (n=34)	108.44 (66.38, 177.13)	6745.13 (5727.50, 7943.55)
No (n=98)	234.94 (191.55, 288.16)	6446.82 (5890.71, 7055.42)
Total	192.48 (130.31, 283.74)	6516 (5695.4, 7603.26)

**Figure 1:**
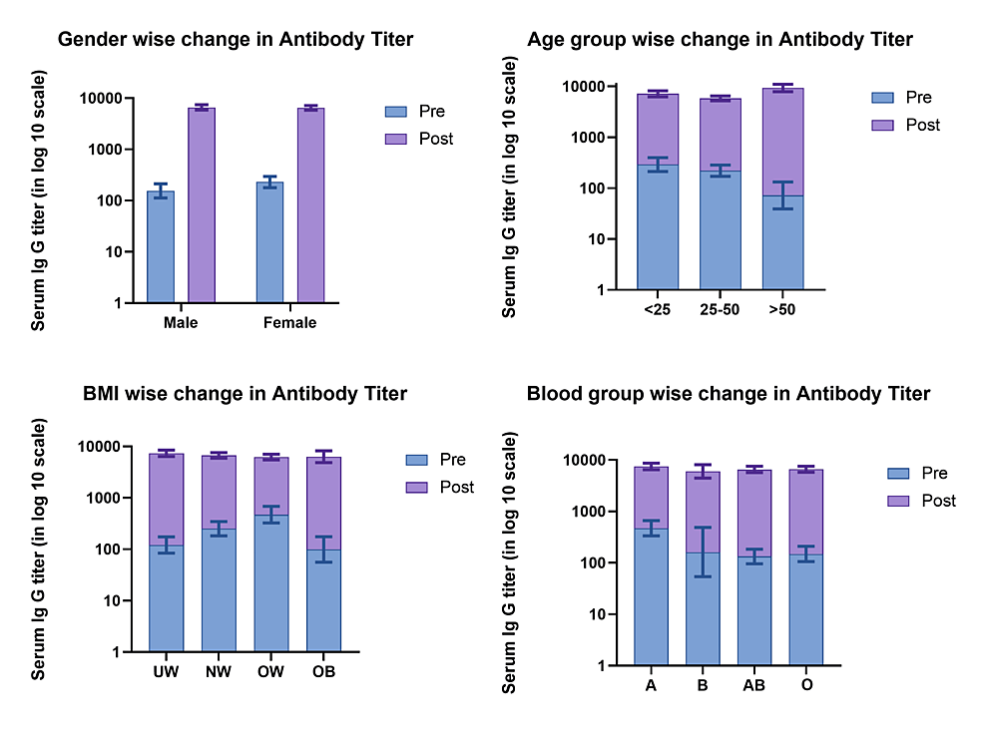
Pre-post anti-spike RBD IgG titer by age, gender, BMI, and blood groups RBD - receptor binding domain, BMI - body mass index

Following the booster dose, there was a substantial increase in antibody levels, rising from a geometric mean titer of 192.48 AU/ml (with a confidence interval of 130.31 to 283.79 AU/ml) to 6516 AU/ml (with a confidence interval of 5695.4 to 7603.26 AU/ml). Table 2 shows the change in antibody titer one month after the booster dose for the different categories of the selected variables. Figure 1 depicts the change in antibody titer in the box plot.

**Figure 2:**
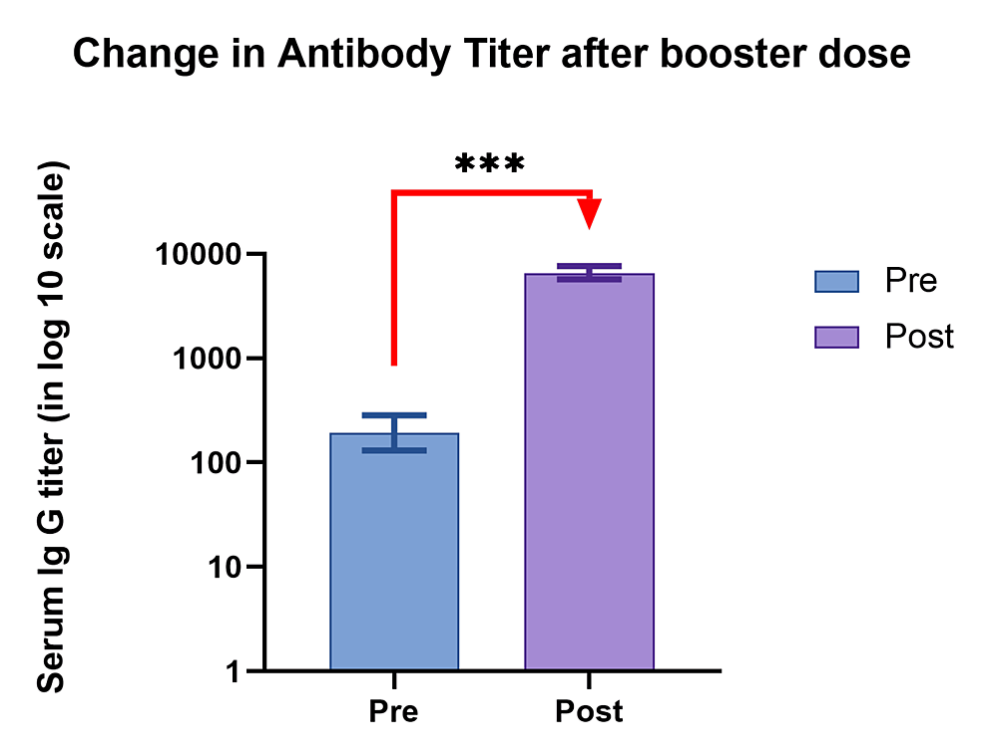
The difference in antibody titer before and one month after a booster dose *** Significant at a p-value of less than 0.0001

It is important to note that the difference in antibody levels before and after the booster dose did not follow a normal distribution. We utilized the Wilcoxon signed-rank test to test the hypothesis regarding this difference. Our analysis revealed a statistically significant difference in antibody levels before and one month after the booster dose. The median difference was 5071 AU/ml, with an interquartile range of 2396.9 to 10477.6 AU/ml, and the p-value was less than 0.0001 (Figure 2).

## Discussion

Our study has shown the excellent immunogenic potential of the ChAdOx1 booster dose in the face of waning immunity. Protection against symptomatic COVID-19 remains effective for at least three months after a single dose of the ChAdOx1 nCoV-19 vaccine and is sustained above baseline levels even after a year [17]. Research has shown that longer intervals between the first and second vaccine doses result in higher antibody levels [18]. However, numerous studies have reported a gradual decline in antibody levels following the initial vaccination [17-21]. Our study also observed seronegativity among 38 participants before the booster dose, indicating a decrease in immunity after nine months of the primary series of vaccines. Interestingly, this decline in immunity was similar for both sexes, consistent with other studies [15]. However, we noted a higher decline in individuals aged 50 or older, aligning with findings from a large UK-based study, suggesting that immunity wanes with age [22].

Our findings indicate a statistically significant increase in antibody titers after the booster, consistent with studies suggesting that a third dose of ChAdOx1 nCoV-19 is well-tolerated and significantly enhances antibody levels [17]. Similar results have also been reported for other COVID-19 vaccines [19,21].

In our study, we observed that changes in antibody levels were similar between males and females [23], in contrast to some studies that reported lower antibody concentrations in males [24]. Additionally, older participants (age ≥ 50) exhibited a more robust immune response, which contrasts with other findings [23,25]. This may be due to recurrent COVID-19 infections naturally boosting immunity, although our study did not consider infection history.

BMI's potential impact on vaccine efficacy has raised concerns, but our study found lower vaccine effectiveness in terms of immune response among underweight individuals. Some studies have found no significant association between BMI and immunogenicity [23]. While comorbidities were associated with reduced vaccine effectiveness in some studies [22], we found similar antibody responses in individuals with and without comorbidities. Further large-scale, community-based studies are needed to explore the relationship between age, sex, BMI, comorbidity status, and antibody responses to the ChAdOx1 nCoV-19 vaccine.

Booster doses have shown promise in preventing severe COVID-19 outcomes, with Barda et al. reporting high effectiveness in preventing hospital admission, severe illness, and COVID-19-related deaths compared to two primary vaccination doses [26]. Other studies have also demonstrated the protective role of third doses against SARS-CoV-2 infection [27]. Moreover, a third dose can induce higher titers of neutralizing antibodies against different variants and boost spike-specific T-cell responses [17]. Robust antibody titers have been linked to reduced disease severity and fewer breakthrough infections [28]. A similarly strong correlation was observed between anti-spike RBD IgG titers and neutralizing antibody titers [29,30]. However, further research is needed to understand the dynamics of antibody concentrations and cellular responses.

While booster doses hold promise, emerging new variants like Omicron present challenges. Some individuals have been infected with Omicron even after receiving a third dose, suggesting significant immune escape [21]. However, studies have reported increased neutralizing antibody responses against Omicron following a booster dose [19]. Booster doses of ChAdOx1 nCoV-19 have shown good tolerability, with few adverse events reported, most of which were mild to moderate in severity [17,25].

Our study has limitations, including a focus on IgG data against the spike protein's receptor-binding domain (RBD) and a one-time measurement of antibody titers after the booster dose. Extended follow-up is needed to assess durability and persistence. Tolerability and adverse events were not within the study's scope, and generalizing the effectiveness of the ChAdOx-1 booster dose requires further population-based research. The study's scope can be expanded to include different current vaccines and booster regimens, potentially enhancing immunity and vaccine effectiveness against evolving variants.

## Conclusions

Our results indicate that giving a booster dose of the ChAdOx1 nCoV-19 vaccine substantially raised the levels of immunoglobulins against the spike protein's receptor-binding domain (RBD). These levels had declined considerably since the initial vaccination. This highlights the importance of administering booster doses on time to maintain stronger and longer-lasting antibody responses. As new variants emerge, our study provides scientific recommendations for periodic booster doses of COVID-19 vaccines to provide optimum protection to our population, especially those with a comorbidity or immunocompromised state.
